# Dental Caries

**Published:** 1884-11

**Authors:** 


### Dental Caries.

The ætiology of this important affection has always been a matter of much difficulty, although it has been closely studied. The more highly civilized races suffer most from it. The character of the food, the use of hot drinks, the overcrowding of the teeth by shortening of the jaws, have all been assigned as causes of the disease, and it is not improbable that all these contribute somewhat to it, but the general opinion has been that it is never entirely traumatic, the condition of the tooth structure itself having an essential relation to the development of decay. Lately, however, considerable attention has been given to the microbe theory of the disease. Several experimenters claim to have shown that acid or alkaline fluids are not capable, in themselves, of producing destruction of dentine, and that it is only under the conditions favorable to the development of micro-organisms that anything analogous to caries will take place. The question can hardly be regarded as settled, although the advocates of the microbe theory are very enthusiastic in defense of it. In its practical bearings the doctrine will not materially modify the rule now in force for the preservation of the teeth—cleanliness.—*The Polyclinic.*

